# Notes on Cars and Garages

**Published:** 1909-06-12

**Authors:** 


### Motoring Notes.

### NOTES ON CARS AND GARAGES.

### British and Foreign Cars: A Comparison.

Several correspondents have recently applied for information and advice upon a matter which is of general interest to medical motorists. This is the comparative merits of the British small car of moderate price and the more expensive foreign-built car of approximately equal size and power. It is, therefore, worthy of note that at the last Olympia Show several continental experts asserted, and with some truth, that the price at which some of the medium-powered chassis are placed upon the market absolutely precludes the use of the finest material and workmanship. They admitted that during the past few years British firms have made enormous progress, and they quite realised that fewer French cars are now being sold in England. At the risk of being thought unpatriotic, I must say that I am inclined to agree with the assertion that, with the exception of a few of our leading manufacturers, we in this country are still a long way behind the French in the matter of automobile design and workmanship. As regards the building of bodies, it is well known that the work of a first-class British coachbuilder cannot be equalled by any foreign firm. In England it is possible to buy medium-powered cars in great variety at prices very much lower than anything that can be purchased abroad. These cars for a year, or possibly longer, will run beautifully, giving absolutely no trouble. But only too often at the end of this period of running they seem to go all to pieces, and thenceforward are a continual source of trouble and expense in repairs and replacements.

### Bad Cheap Cars.

There is no such thing as a really cheap car, because if it is very cheap in the beginning it will sooner or later prove to be very expensive. First-class materials and first-class workmanship are not, and never can be, cheap, and the sooner the British buyer understands this the better it will be for him in the long run. For instance, it is not so long ago that a British car, made by a well-known manufacturer, ran practically 15,000 miles non-stop, averaging something over twenty miles to the gallon. This car was then dismantled under supervision, and it was discovered that a little over £2 would cover all the necessary replacements. Now, 15,000 miles represents the distance travelled by the average motorist in three years, and although this car was not cheap in the first instance, it was most certainly cheap in the long run. Nothing causes more annoyance and disappointment than a car which, after doing good service for the first twelve months, continually goes wrong in the second year. It destroys all the pleasure of motoring, because each journey is begun with the certainty that sooner or later, somewhere or another, a breakdown is inevitable. The consequence is that at last the breaking point of patience is reached, and the car is advertised for sale, and probably disposed of for a mere fraction of its original cost. Whereas, had a really first-class car been bought in the first instance, this would have done honest, reliable work for several years, with a maximum of comfort and satisfaction; and when its owner decided to replace it by a more modern type it would have realised a good price in the second-hand market as a "going concern."

### Garages and Garage Skates.

Nothing can be nicer than a well-appointed garage, though, unfortunately, all are not rich enough to afford this luxury; and, moreover, sufficient space is not always available. The problem is usually how to make an existing outbuilding or coach-house serve one's purpose. The trouble generally arises when one wishes to manœuvre the car inside the shed or turn it within a small space. A turntable, though undoubtedly a great labour-saver, is exceedingly expensive, and beyond the financial resources of the average owner.

Garage skates, as they are styled, are inexpensive, and by their use a car can be turned in any direction. A pair are simply put on the wheels, and the car is pulled round with a very little expenditure of energy. A device such as this frequently saves broken tail lamps and damaged wings, minor accidents which are inseparable from turning in a small space. However small the garage, always make a point of fitting as large a door as possible. The best design is a door on rollers extending the whole breadth of the garage. If a pit is fitted, make it a rule never to leave it uncovered. Otherwise the car will be driven into it some night.

### The Clutch and the Fly-wheel.

The importance of having a good clutch cannot be over-estimated, since an inferior clutch renders driving a source of pain and grief. It certainly seems as though the art of clutch design has improved but little during the last few years. On some of the old cars—Panhards, Charrons, etc.—the leather-faced cone clutch seldom, if ever, gave a moment's trouble, and these clutches were nearly always smoothest in engagement. The experience of many drivers with metal-disc clutches, other than those of high-class cars, has been far from happy. Sooner or later they have given rise to trouble. Moreover, these clutches need accurate and skilful adjustment, and the lubricant must be chosen with the utmost care, or dire results will follow. Provided a leather clutch is of proper design, it should give no trouble so long as care is taken to dress it occasionally with cotton or castor oil, and the possible necessity for replacement of the leather is considered at the end of twelve months. In addition to the type of clutch, prospective buyers should pay attention to the fly-wheel, insisting that a sufficiently large fly-wheel is used, since on this depends largely the smooth and flexible running of the car, especially when of the single-cylinder type; and many makers who build this type of car are now fitting much larger fly-wheels than formerly.

